# Follicle Online: an integrated database of follicle assembly, development and ovulation

**DOI:** 10.1093/database/bav036

**Published:** 2015-04-29

**Authors:** Juan Hua, Bo Xu, Yifan Yang, Rongjun Ban, Furhan Iqbal, Howard J Cooke, Yuanwei Zhang, Qinghua Shi

**Affiliations:** ^1^Molecular and Cell Genetics Laboratory, The CAS Key Laboratory of Innate Immunity and Chronic Disease, Hefei National Laboratory for Physical Sciences at Microscale, School of Life Sciences, University of Science and Technology of China, Hefei 230027, China, ^2^Department of Biochemistry and Molecular Biology, School of Basic Medical Sciences, Anhui Medical University, Hefei 230032, China, ^3^Center for Reproductive Medicine, Anhui Provincial Hospital Affiliated to Anhui Medical University, Hefei 230001, China, ^4^Department of Statistics, University of Kentucky, Lexington, KY 40506, USA, ^5^Collaborative Innovation Center of Genetics and Development, Fudan University, 2005 Songhu Road, Shanghai 200438, China, ^6^Hefei Institutes of Physical Science, Chinese Academy of Sciences, Hefei 230031, China and ^7^Institute of Pure and Applied Biology, Bahauddin Zakariya University Multan, 60800, Pakistan

## Abstract

Folliculogenesis is an important part of ovarian function as it provides the oocytes for female reproductive life. Characterizing genes/proteins involved in folliculogenesis is fundamental for understanding the mechanisms associated with this biological function and to cure the diseases associated with folliculogenesis. A large number of genes/proteins associated with folliculogenesis have been identified from different species. However, no dedicated public resource is currently available for folliculogenesis-related genes/proteins that are validated by experiments. Here, we are reporting a database ‘Follicle Online’ that provides the experimentally validated gene/protein map of the folliculogenesis in a number of species. Follicle Online is a web-based database system for storing and retrieving folliculogenesis-related experimental data. It provides detailed information for 580 genes/proteins (from 23 model organisms, including *Homo sapiens, Mus musculus, Rattus norvegicus, Mesocricetus auratus, Bos Taurus, Drosophila* and *Xenopus laevis*) that have been reported to be involved in folliculogenesis, POF (premature ovarian failure) and PCOS (polycystic ovary syndrome). The literature was manually curated from more than 43 000 published articles (till 1 March 2014). The Follicle Online database is implemented in PHP + MySQL + JavaScript and this user-friendly web application provides access to the stored data. In summary, we have developed a centralized database that provides users with comprehensive information about genes/proteins involved in folliculogenesis. This database can be accessed freely and all the stored data can be viewed without any registration.

**Database URL:** http://mcg.ustc.edu.cn/sdap1/follicle/index.php

## Introduction

Folliculogenesis consists of three main processes including primordial follicle assembly, follicle development and ovulation (1–3). It is the progression of number of small primordial follicles into large preovulatory follicles that enters the menstrual cycle (4, 5). Folliculogenesis begins with primordial follicle assembly (during the perinatal period in human, mouse and rat) during which the oocytes that survive the process of germ cell cluster breakdown are individually surrounded with squamous pre-granulosa cells (6–11). The recruitment (or the initial growth) of primordial follicles is defined by a dramatic enlargement of the oocyte itself, accompanied by proliferation and differentiation of the surrounding granulosa cells (11–14). Ovulation is the last step of folliculogenesis, which involves the breakdown of a mature ovarian follicle to release a fertilizable oocyte (14–18).

The ultimate role of the folliculogenesis, a tightly regulated process that lasts for two to five decades in women, is to produce a healthy egg that will transmit genes to the next generation (18–23). Inappropriate coordination of these events will result in serious ovarian pathologies such as POF (premature ovarian failure) and PCOS (polycystic ovary syndrome) (24, 25). In humans, POF is defined as an ovarian defect characterized by absence of menarche (primary amenorrhea) or premature depletion of ovarian follicles/arrested folliculogenesis before the age of 40 years (secondary amenorrhea, 25). POF affects ∼1 in 10 000 women by the age of 20 (0.01%), 1 in 1000 women by the age of 30 (0.1%) and 1 in 100 women by the age of 40 (1%, 24). PCOS is another common female endocrine disorder that affects ∼5–10% of women of reproductive age (12–45 years) (24). These two diseases are considered to be the leading causes of female subfertility and the most frequent endocrine problems in women of reproductive age (24). Currently there are no effective treatments for these ovarian pathologies and in many cases, POF and PCOS are generally diagnosed only when women have already lost their fertility (24, 25).

Although the physiological function of folliculogenesis is well defined, the mechanisms controlling this process are not properly understood. Until recently, the tools available to reproductive biologists to determine the functions of genes involve in mammalian folliculogenesis were based on the study of homologous genes in lower model organisms and the production of genetically modified mice with targeted disruptions of genes of interest (26, 27). These studies not only suggested that which molecules may be involved in the folliculogenesis in human but they were also able to highlight specific candidate molecules that can be used as targets for potential drugs used to treat ovarian pathologies (26, 27). However, information about the genes/proteins involved is folliculogenesis was scattered across thousands of publications and extraction of relevant functional information from various resources is a significant task. Thus, an integrated database of the existing resources regarding folliculogenesis was urgently required.

By searching literature, we manually collected unique proteins from 23 species with experimentally verified functions during folliculogenesis. This led us to the development of an integrated and searchable database, Follicle Online. We present here the database (Follicle Online) of genes experimentally linked with folliculogenesis (also PCOS and POF). This online service is implemented in PHP + MySQL + JavaScript. Follicle Online can provide potentially orthologous genes for these genes/proteins reported in Follicle Online from seven model organisms, including *Homo sapiens, Mus musculus, Rattus norvegicus, Macaca mulatta, Danio rerio, Bos taurus* and *Gallus gallus*. Follicle Online will be regularly updated as soon as new genes/proteins regulating folliculogenesis (also PCOS and POF) will be reported. It possess a comprehensive set of features, which allows users to explore different aspects of functionality of the folliculogenesis genes indexed in Follicle Online and provides important information required for further experimentally or bioinformatics analysis of these genes.

## Materials and methods

**Figure 1.**
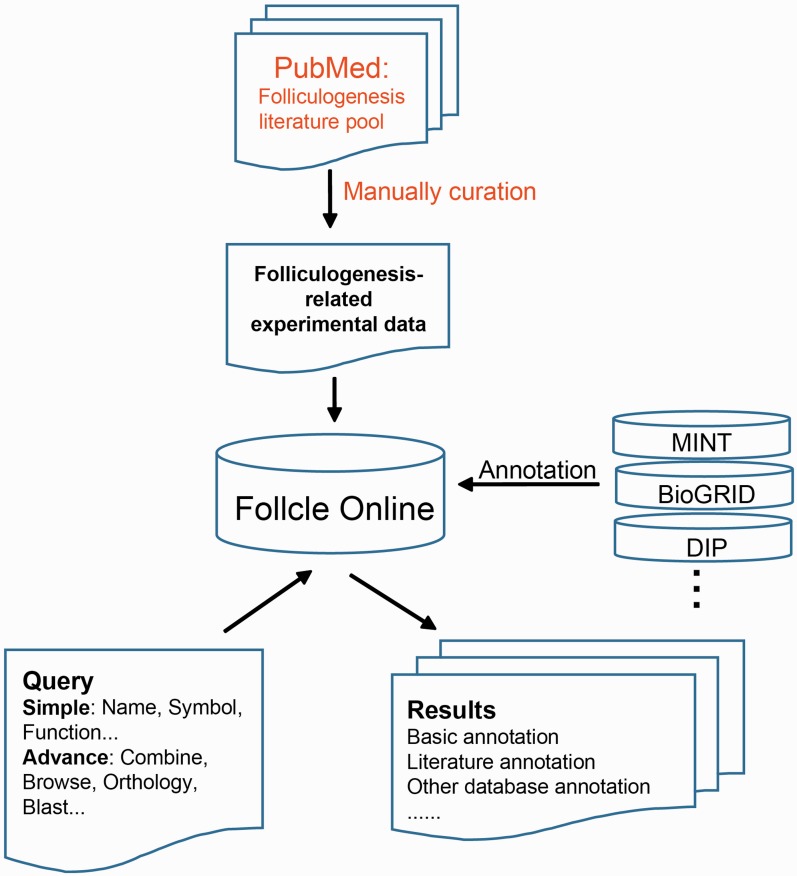
Follicle Online Database scheme.

The general process of data collection and annotation for Follicle Online is illustrated in Figure 1.

### Data generation

As data quality is critical for any database, we searched PubMed with specific key words for different stages of folliculogenesis and have collected experimentally identified functional genes/proteins from more than 43 000 published articles (till 1 March 2015). The inclusion criterion was that only those genes/proteins will be represented in database the function of who has been experimentally verified. Genes/Proteins for which only expression information was available were excluded from Follicle Online.

To search for follicle assembly genes/proteins in PubMed, we used the keywords ‘primordial follicle endowment’, ‘primordial follicle formation’ and ‘primordial follicle assembly’. For proteins involve in follicle development, we used the keywords ‘primary follicle’, ‘follicle activation’, ‘follicle transition’, ‘follicle recruitment’, ‘secondary follicle’, ‘preantral follicle’, ‘preantral folliculogenesis’, ‘follicle development’, ‘antral follicle formation’, ‘preovulatory follicle’ and ‘follicle growth’ as queries, as these 11 keywords can cover the entire process of follicular growth. In addition, for genes/proteins associated with ovulation, we used ‘ovulation’ as keyword to search PubMed. To avoid any missing data, we also searched by using the keywords ‘cumulus expansion’ and ‘luteinization’. For PCOS and POF, we used ‘polycystic ovary syndrome’ and ‘premature ovarian failure’ as keywords to search in PubMed, respectively. In total, we collected 578 unique Follicle Online genes/proteins from 23 organisms (Table 1).

**Table 1.** Follicle Online data types and their corresponding numbers

Species (number)	A	D	O	PCOS	POF	Multi-process
Mus musculus (207)	12	104	67	2	0	22
Homo sapiens (175)	2	18	7	118	24	6
Rattus norvegicus (66)	3	43	8	6	0	6
Bos taurus (33)	0	17	13	0	0	3
Sus scrofa (28)	1	11	14	0	0	2
Others (71)	11	29	22	0	4	5
Total (580)	23	222	131	126	28	44

A, follicle assembly; D, follicle development; O, ovulation. PCOS, polycystic ovary syndrome; POF, P remature ovarian failure.

### Protein annotation

Genes/Proteins in Follicle Online were annotated as follows: (i) the basic information [e.g. name/synonyms, protein sequences, nucleotide sequences, PI (isoelectric point) and Mw (molecular weight)] of the genes/proteins were annotated referring to GenBank and UniProt Knowledgebase (Release 2015_01); (ii) detail description of the genes/proteins [subcellular location, folliculogenesis functional stage and disease type] were provided from the experimentally verified data, also the result figures and abstracts of literatures (PubMed) about genes/proteins reported in Follicle Online are provided; (iii) the PPI (protein–protein interaction) information, the orthologous of each genes/proteins, functional domain, structural domain and the GO term were also provided. The PPI information was integrated from several major public databases, such as HPRD (Release 9), BioGRID (Verision 3.2.119), DIP (Released 20130131), MINT (Downloaded 20130520), IntAct (Downloaded 20140329) and STRING (Version 9.1) (28–31). The redundant PPIs were removed and the cleared PPIs were divided into two parts, the experiment verified and predicted PPIs.

### Microarray data collection

To collect the expression information for genes/proteins reported in Follicle Online database, the ArrayExpress database (http://www.ebi.ac.uk/arrayexpress) was used as a resource. We screened the whole ArrayExpress database and downloaded whole transcript microarray experiment datasets related with folliculogenesis from 18 mouse models by using Affymetrix GeneChip Mouse Genome 430 2.0 platform. The mRNA expression information of genes reported in Follicle Online database were extracted from these microarray data and presented in the web page.

## Results

### User interface—simple and advanced search

**Figure 2.**
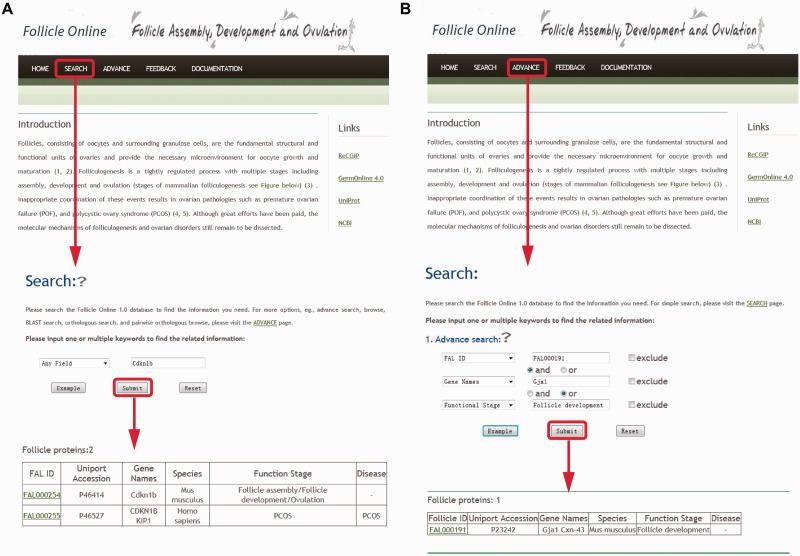
**(A)** Screen shots of user interface—simple search. Simple search query using one of the six options provided e.g. Gene Name. **(B)** Screen shots of user interface—advance search. The search mode is an advanced search with multiple parameters that permits retrieval of very fine subsets of data.

Follicle Online provides a search engine for users to search the genes/proteins of their interest. The search option (http://mcg.ustc.edu.cn/sdap1/follicle/follado.php) provides an interface for querying the Follicle Online database with one or more keywords such as genes/proteins names, UniProt ID or Follicle Online ID, functional stage and function etc (Figure 2A).

There is also an ‘Advance search’ option in Follicle Online that allows multiple fields search simultaneously (Figure 2B). Search options includes: (i) advance search where users can use relatively complex or combination of keywords to locate the precise information. The interface of search-engine permits the querying by different annotation fields and the queries are linked by three operators (‘and’, ‘or’ and ‘exclude’); (ii) Browsing, instead of searching for a specific protein, all entries of Follicle Online database could be listed by species name, functional stage. The human reproductive tract diseases are also included in this database as a dropdown menu under the ‘Disease’ section. Users can choose the disease of their interest from this menu and can explore it further; (iii) BLAST search; this option was designed to quickly find all the related information about a gene/protein reported in Follicle Online database. For this purpose, the blast program in NCBI BLAST is included in Follicle Online database. Users can input a protein sequence in FASTA format for searching identical or homologous proteins; (iv) orthologous search, user can specifically browse the orthologous information for a protein by providing the genes/proteins names.

### User interface results

**Figure 3.**
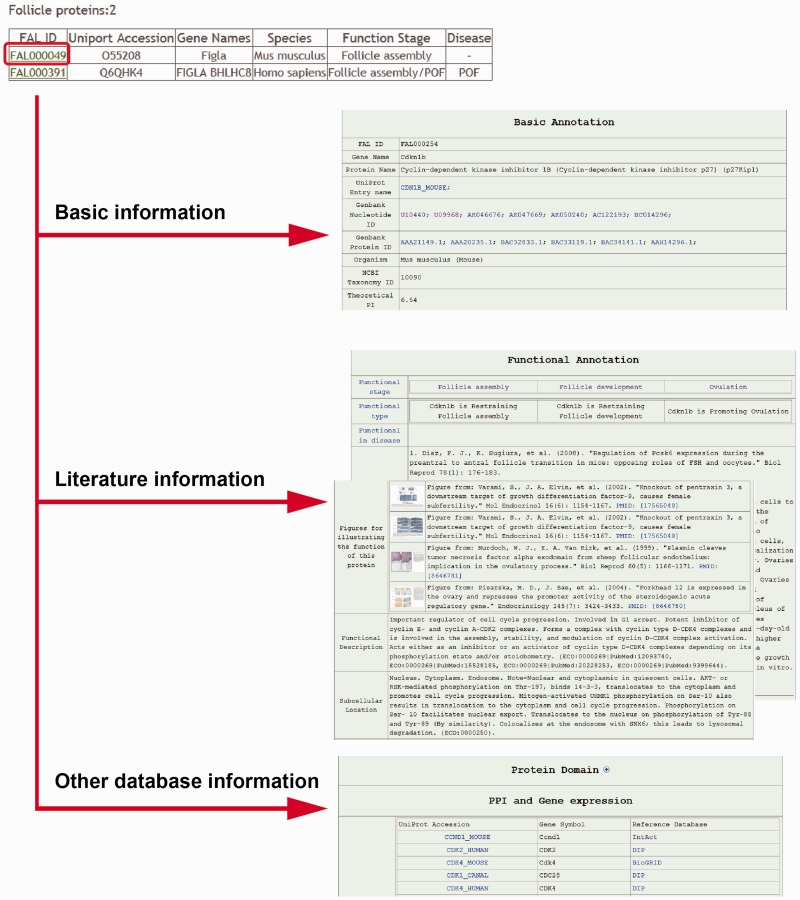
Screen shots of user interface—result page. Collage of some of the results produced via clicking the ‘Browse’ button in both ‘Simple’ and ‘Advanced’ search options.

The results for each search returns information in tabular format (Figure 3). The results for each search are displayed in three part: (i) the basic information [e.g. gene name, nucleotide and protein sequences, Mw (molecular weight)]; (ii) the literature information [functional stage, disease type (follicle endowment, growth, ovulation, PCOS or POF), regulation type (positive or negative), figures and abstracts from the reference literature, functional description, subcellular location of FAL proteins, tissue specificity of FAL proteins and GO annotation]; (iii) the other database information [Protein domain organization, PPI information and mRNA expression information from folliculogenesis related microarray data].

### Feedback

Users are welcome to review Follicle Online records. They can revise the Follicle Online records as well as can submit novel proteins or proteins with novel function in folliculogenesis or in any disorder associated with folliculogenesis. The contribution from the users is highly encouraged.

## Discussion

Folliculogenesis is a multi-faceted and tightly regulated process that includes primordial follicle assembly, follicle development and ovulation (1–6). In recent years, many critical molecules involved in folliculogenesis have been identified, mainly through the use of genetically modified mouse models. The data obtains from these models provide clues about abnormal reproductive conditions in human (7–9). Multiple genes have been reported to regulate folliculogenesis and fertility in animals and they also play role in human reproduction because of evolutionary conservation between species (10–12). To effectively analyse data generated in various experiments, it is necessary to manage this data in an organized manner.

Follicle Online is a database for storing and retrieving all the folliculogenesis-related experimental data. The core of Follicle Online consists of genes/proteins involved in folliculogenesis with their experimentally confirmed functional role. All these genes/proteins identified in *Homo sapiens, Mus musculus* and *Rattus norvegicus* were submitted to a GO enrichment analysis. Using the proteome as the background, we statistically calculated the represented biological processes, molecular functions and cellular components in Follicle Online genes/proteins distribution (*P* < 0.05). The top 10 most enriched GO terms in each category are shown in Supplementary Tables S1–S3. We then enriched the biological pathway of 580 Follicle Online genes/proteins from Kyoto Encyclopedia of Genes and Genomes (KEGG) pathway. The enriched pathway entries in each category are represented in Supplementary Tables S4–S6. This analysis further illustrates the essential roles played by genes/proteins reported in Follicle Online during folliculogenesis and facilitates the users to uncover the undiscovered functions of these genes/proteins from *Homo sapiens, Mus musculus* and *Rattus norvegicus* documented in Follicle Online.

**Figure 4.**
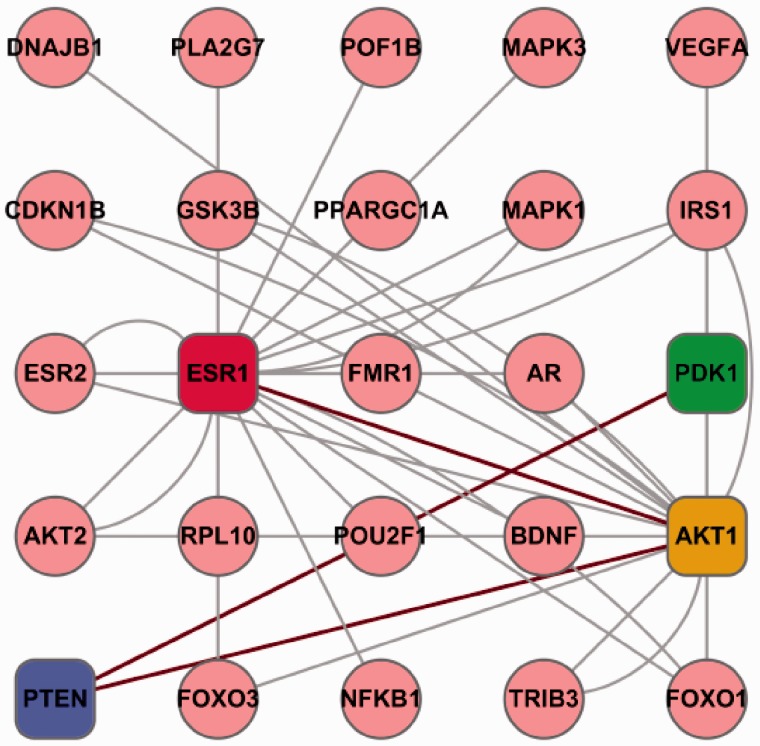
An example of potential protein network of folliculogenesis. The proteins in square frame (ESR1, PDK1, AKT1 and PTEN) are reported to participate in folliculogenesis. The proteins in circle are predicted to interact with the four proteins (in square boxes) either directly or indirectly. Red lines represents the experimentally verified interactions. Although the grey line shows the predicted interactions of proteins by our database.

Folliculogenesis must be, in part, dictated by networks of genes expressed in the ovary. Thus, PPI information for proteins reported in Follicle Online was critical for understanding the mechanisms of folliculogenesis. Combined with experimentally validated and computationally predicted PPIs, we constructed a potential protein network for folliculogenesis with 111 proteins having 208 interactions (Figure 4). When we carefully analysed the scientific literature, we found that some of our listed interactions have been reported to be involved in folliculogenesis. For example, John *et al*. (32, 33) showed that the PI3K pathway has a key role in the initiation of follicle growth, while it has been documented that oocyte-specific deletion of Pten in mice resulted in POF (34). Female mice deficient for Pten interaction proteins Akt1 and PDK1 also exhibited significant primordial follicle loss and POF (35, 36). Esr1, the Akt1 interaction proteins also have been shown to take part in folliculogenesis (37). Moreover, our results also indicated a number of interesting interactions whose function in folliculogenesis is unknown at the moment. Hence, to explore the role of these proteins in folliculogenesis would be very interesting for the groups working in this research field.

Follicle Online is specifically focused on the genes/proteins involved in folliculogenesis and is distinct from the published online public databases like Ovarian Kaleidoscope that is providing about all the proteins expressing in ovary (38). The first differentiating feature is that the data included in Follicle Online are from 23 model organisms including *Homo sapiens, Mus musculus, Rattus norvegicus*. However, the data in the Kaleidoscope database is only from species such as *Homo sapiens, Mus musculus, Rattus norvegicus* and *Bos taurus* genomics. All genes/proteins reported in Follicle Online are retrieved from experiments that have identified their unique functions during folliculogenesis. After data acquisition, the information on the functional genes, including location, biological processes, regulation mode reported in the literature, was manually curated from more than 40 000 research articles retrieved from PubMed (till 1 March 2014). Finally, the genes/proteins functional domains and protein interactions of these proteins reported in Follicle Online was also added as unique features of this database.

In summary, we have developed a database (Follicle Online) that provides a comprehensive platform to get the detailed experimentally validated information regarding the 580 genes/proteins involved in folliculogenesis, POF and PCOS. Follicle Online can be accessed via http://mcg.ustc.edu.cn/sdap1/follicle/index. All the data stored in the database can be accessed without any registration. Follicle Online will aid researchers to have a comprehensive understanding of complex biological mechanisms associated with folliculogenesis that will pave the way for the treatment of various disorders associated with folliculogenesis.

## Supplementary Data

Supplementary data are available at *Database* Online.

## Funding

This work was supported by the National Basic Research Program (2013CB947902, 2012CB944402 and 2013CB945502) of China (973), the National Natural Science Foundation of China (31301227), Major Program of Development Foundation of Hefei Center for Physical Science and Technology (2014FXZY003). Research of BSKY (0108035101) from Anhui Medical University, Natural Science Foundation of Anhui Provincial of China (1308085QH131).

*Conflict of interest*. None declared.
